# Risk Factor Clusters and Cardiovascular Disease in High-Risk Patients: The UCC-SMART Study

**DOI:** 10.5334/gh.897

**Published:** 2021-12-21

**Authors:** Emily I. Holthuis, Frank L. J. Visseren, Michiel L. Bots, Sanne A. E. Peters, on behalf of the UCC-SMART study group

**Affiliations:** 1 Julius Center for Health Sciences and Primary Care, University Medical Center Utrecht, Utrecht University, Utrecht, NL; 2 Department of Vascular Medicine, University Medical Center Utrecht, Utrecht, NL; 3 The George Institute for Global Health, Imperial College London, London, UK; 4 The George Institute for Global Health, University of New South Wales, Sydney, AU

**Keywords:** Prevalence, clustering, cardiovascular risk factors, secondary prevention

## Abstract

**Background:**

Clustering of vascular risk factors, i.e., the co-existence of two or more risk factors, has been associated with a higher risk of cardiovascular disease (CVD) in the general population. This study aims to firstly, examine patterns of clustering of major cardiovascular risk factors in high-risk patients and their relation with the risk of recurrent cardiovascular disease and all-cause mortality. Secondly, to assess which combinations are associated with the highest risk of CVD and all-cause mortality and to study population attributable fractions.

**Methods:**

A total of 12,616 patients from the Utrecht Cardiovascular Cohort – Second Manifestations of ARTerial diseases (UCC-SMART) study consisting of patients with or a high risk to develop cardiovascular disease were studied. We constructed sixteen clusters based on four individual modifiable risk factors (hypertension, dyslipidemia, current smoking, overweight). Patients were followed from September 1997 to March 2017. Cox proportional hazard models were used to compute adjusted hazard ratios for CVD risk and all-cause mortality and 95% confidence intervals for clusters, with patients without any risk factor as reference group. The population attributable fractions (PAFs) were calculated. Subgroup analyses were conducted by age and sex.

**Results:**

During a mean follow-up period of 8.0 years, 1836 CVD events were registered. The prevalence of patients with zero, one, two, three, and four risk factors was 1.4, 11.4, 32.0, 44.8 and 10.4%. The corresponding hazard ratios (HR) for CVD risk and all-cause mortality were 1.65 (95% CI 0.77; 3.54) for one risk factor, 2.61 (1.24; 5.50) for two, 3.25 (1.55; 6.84) for three, and 3.74 (1.77; 7.93) for four risk factors, with patients without any risk factor as reference group. The PAFs were 6.9, 34.0, 50.1 and 22.2%, respectively. The smoking-hypertension-dyslipidemia combination was associated with the highest HR: 4.06 (1.91; 8.63) and the hypertension-dyslipidemia combination with the highest PAF: 37.1%.

**Conclusion:**

Clusters including smoking and hypertension contributed to the highest risk of CVD and all-cause mortality. This study confirms that risk factor clustering is common among patients at high-risk for CVD and is associated with an increased risk of CVD and all-cause mortality.

## Background

Cardiovascular disease (CVD) is a major contributor to morbidity and mortality, accounting approximately for one third of all deaths globally [1]. In 2015, around 17.9 million people worldwide died from CVD [2]. The cardiovascular burden is responsible for a substantial share of total health costs and reduced quality of life in society. In high-income countries, CVD mortality is decreasing, due to improved primary and secondary prevention measures [3,4]. Worldwide the incidence of CVD is rising, which can be explained by the high CVD burden in lower- and middle income countries, the overall aging of the population and the increasing prevalence of CVD risk factors [5].

The most important modifiable CVD risk factors are obesity, smoking, high blood pressure, dyslipidemia, and type 2 diabetes [6]. These risk factors commonly co-exist, especially at older age and are thus likely to cluster in an individual [7,8]. For example, being overweight is often present with other traditional risk factors such as hypertension or dyslipidemia. According to previous studies in the general population, the co-existence of two or more risk factors, i.e., clustering, is associated with an increased risk of cardiovascular disease [9,10,11]. While all combinations of risk factors are related to an increased CVD risk, the magnitude of the relation varies across clusters [8,10].

Insights into clustering patterns is relevant for the individual patient, physicians and healthcare providers as this might tailor the use of management and preventive treatment approaches, especially since some risk factor combinations are more atherogenic than others, clarifying the variation in CVD prognosis among high-risk patients. The relationship between individual risk factors along with risk factor clusters and CVD risk is well described in studies in the general population. Yet, insights on clustering patterns in secondary prevention are limited to the metabolic syndrome (MetS) and do not include broader cardiovascular risk factor clusters [12,13].

In this study, we set out to study the prevalence of risk factor clusters, their relation with risk of CVD and all-cause mortality, and population attributable fractions in high-risk patients.

## Methods

Data from 12,616 patients enrolled in the UCC-Second Manifestation of ARTerial disease (SMART) study from September 1997 to March 2017 were used. A detailed description of the protocol and objectives has been described previously [14,15]. In short, UCC-SMART is an ongoing prospective cohort study of patients, aged 18–79, newly referred to the University Medical Center Utrecht (UMCU) with either a history of clinically manifest atherosclerotic vessel disease or risk factors for CVD (e.g. dyslipidemia, smoking, hypertension, diabetes). All patients are at increased risk for (recurrent) cardiovascular disease and thus well reflect a high-risk population [14]. Vascular disease is defined as coronary artery disease (CAD), peripheral artery disease (PAD), cerebrovascular accidents (CVA; including ischemic and hemorrhagic stroke) and abdominal aortic aneurysm (AAA). Patients not proficient in Dutch, with a life expectancy of less than two years, terminal malignant disease, dependent in daily activities (ranking scale >3) and those referred back to the referring specialist immediately after one visit were not enrolled in the study. The study was approved by the Medical Ethics Committee of the UMCU. All patients gave written informed consent [14,15].

### Cardiovascular risk factors

We examined four risk factors; smoking, overweight, hypertension and dyslipidemia, which were studied as individual or combinations (clusters) of risk factors. Smoking status was determined from self-reported questionnaires at inclusion and was defined as current (non) smoking. Overweight was defined as a BMI ≥ 25 kg/m^2^. Blood pressure was measured bilaterally at the brachial arteries with the use of an automatic blood pressure monitor (Microlife Watch BP office). Following the first measurement, it was determined which arm had the highest systolic blood pressure. Thereafter, the average systolic and diastolic blood pressure was taken from two additional measurements from the arm with the highest blood pressure [14,15]. Hypertension was defined as systolic blood pressure ≥140 mmHg and/or diastolic blood pressure ≥90 mmHg in office blood pressure measurements and/or prescribed antihypertensive drugs. To determine lipid levels, a venous blood sample was taken. Plasma total cholesterol was measured with the use of commercial enzymatic dry chemistry kits (Johnson and Johnson). Plasma HDL-Cl was evaluated with commercial enzymatic kit (Boechringer-Mannheim) following precipitation of LDL and VLDL with sodium phosphotungstate magnesium chloride [14]. Dyslipidemia was defined as non-HDL levels (total cholesterol minus HDL-C) >2.6 mmol/L and/or the prescription of lipid lowering drugs [16]. Diabetes mellitus was defined as referral diagnosis of diabetes, self-reported diabetes (use of glucose-lowering medication), known history of diabetes mellitus at the time of enrolment or when fasting plasma glucose was ≥7 mmol/L [13]. Renal function was estimated with Modification of Diet in Renal Disease (MDRD) and classified as impaired when eGFR (estimated glomerular filtration rate) <60 ml/min/1.73 m [17]. The prevalence of diabetes mellitus and impaired renal function was 18.2% and 12.7% however, when combined with other risk factors in clusters, the prevalence was relatively too low to be included in the clusters and to estimate their relation with cardiovascular events.

Two aspects regarding the risk factor clusters definitions should be clarified. Firstly, regarding obesity, it could be that some of the adverse effects of obesity are due to its downstream metabolic effects on risk factors. However, obesity can increase CVD morbidity and mortality directly. For example, through obesity-induced and functional adaptations of the cardiovascular system to accommodate excess body weight or through the adipokine effects on inflammation and vascular homeostasis. Also, previous studies in primary prevention on risk factor clusters and CVD events also included obesity in their clusters) [8,10]. To facilitate comparability between studies, we therefore also included obesity in our clusters. Secondly, the hypertension and dyslipidemia definitions include medication. The blood pressure and lipid-lowering medication might be prescribed to treat an elevated risk factor level or because the patient has a high total risk for CVD event. We do not have information on lipid and blood pressure levels before medications were initiated nor on the reason for initiation. However, the current definitions are consistent with the definitions in other studies in SMART [14,15].

### Study endpoint

As described previously [14,15], during follow-up, information on hospitalization and outpatient clinic visits was obtained through biannual questionnaires. Death was reported by relatives, the general practitioner or treating specialist. All events were independently evaluated by three members of the SMART study endpoint committee. The primary outcome was a composite of major CVD events [myocardial infarction (MI), stroke and vascular mortality] and all-cause mortality (International Classification of Diseases 10^th^ Revision: I20-I25, I60-I69). MI was defined as at least two of the following criteria: (a) chest pain for at least 20 minutes, not disappearing after administration of nitrates; (b) elevation of the ST-segment >1 mm in two following leads on an electrocardiogram or a left bundle branch block; and (c) cardiac enzyme elevation (troponin above clinical cut-off value or creatinine kinase of at least two times the normal value and a myocardial band fraction >5% of the total creatinine kinase). Sudden cardiac death was also considered as an MI. Stroke was defined as definite stroke when the relevant clinical features were present for ≥24 hours causing an increase in impairment of ≥1 grade on the modified ranking scale as well as a new cerebral infarction on the CI or MRI. Stroke was defined as probable stroke when the relevant clinical features where present for ≥24 hours causing an increase in impairment of ≥1 grade on the modified ranking scale, yet with no new (haemorrhage) cerebral infarction on the CT or MRI [15]. Vascular mortality was defined as death caused by MI, stroke, congestive heart failure, rupture of abdominal aortic aneurysm and vascular death from other causes. All-cause mortality, also part of the study endpoint, was defined as death from any cause [15]. The study endpoint included both recurrent and new cases of CVD. The period between patient inclusion and first (recurrent) cardiovascular event, death from any cause, loss to follow-up or the predefined date of March 2017 was defined as the follow-up duration. In total, 880 patients (7.0%) were lost to follow-up because of relocation or discontinuation of their participation.

### Statistical analyses

Baseline characteristics were described as mean ± SD for continuous variables and as frequencies and percentages for categorical variables. Prevalence estimates of the clusters are given as percentages with corresponding 95% confidence limits.

Cox proportional hazard models were used to estimate hazard ratios (HR) and 95% confidence intervals (CIs) for the risk of developing CVD and all-cause mortality associated with risk factor clusters. The reference group are patients with none of the four risk factors. Analyses were adjusted for age and sex. Subgroup analyses were conducted by age (cutoff was based on the median of 60 years) and sex. A complete case analysis was performed since the proportion of missing data on risk factors was 1.1%, and thus multiple imputation would not majorly affect the estimates [18]. The population attributable fraction (PAF) was also calculated with the following formula: PAF = \frac{{{\rm{prevalence}} * {\rm{(HR}} - 1{\rm{)}}}}{{1 + prevalence * (HR - 1)}} [19]. The PAF is the proportion of the CVD diseases that could be prevented by elimination of a causal risk factor or cluster in this high-risk population. All constructed risk factor clusters were mutually exclusive in calculating the hazard ratios and the corresponding confidence intervals. Risk factor clusters were not mutually exclusive in calculating the PAFs. As such, the prevalence of a risk factor was the sum of the prevalences of the clusters that included the respective risk factor. For example, the prevalence of risk factor ‘dyslipidemia’ was based the dyslipidemia prevalence as an individual risk factor in addition to the prevalence of the smoking-dyslipidemia, dyslipidemia-overweight, smoking-hypertension-hyperlipidemia, hypertension-dyslipidemia-overweight and smoking-hypertension-hyperlipidemia-overweight clusters. All data was analyzed with R Version 1.1.456.

## Results

### Baseline characteristics

The mean age of the 12,616 patients was 56.3 ± 12.5 years and 65.5% were men. In total, 28.1% of the patients were current smokers at inclusion and the average BMI was 26.9 ± 4.4. The mean systolic and diastolic blood pressure (mmHg) were 141 ± 22 and 83 ± 13, respectively, and 46.1% had a prescription of blood pressure lowering medication. The average non-HDL (mmol/L) was 3.8 ± 1.4. Overall, 43% had a prescription for lipid lowering medication (Table 1). Baseline characteristics for the reference group and for patients with one, two, three and four risk factors were also included in Table 1.

**Table 1:** Baseline characteristics of the UCC-SMART study included in the analyses (N = 12,616).

Variable	Overall N = 12,616	Reference group N =174	1 Risk factors N = 1422	2 Risk factors N = 3997	3 Risk factors N = 5586	4 Risk factors N = 1300

**Age (years)**	56.3 ± 12.5	49.8 ± 15.9	53.1 ± 14.2	56.3 ± 13.0	57.7 ± 11.5	54.8 ± 10.6
**Men**	8263 (65.5)	102 (58.6)	866 (60.9)	2584 (64.6)	3734 (66.8)	901 (69.3)
**Body mass index (kg/m^2^)**	26.9 ± 4.4	22.3 ± 1.8	23.3 ± 2.6	25.3 ± 3.8	28.4 ± 4.3	29.5 ± 3.9
**High body mass index (BMI > 25 kg/m^2^)**	8210 (65.1)	0 (0)	173 (12.2)	1761 (44.1)	4888 (87.5)	1300 (100)
**History of CVD**						
**CAD**	3865 (30.7)	72 (41.4)	458 (32.2)	1280 (32.1)	1683 (30.1)	350 (26.9)
**PAD**	1133 (9)	3 (1.7)	68 (4.8)	286 (7.2)	57.8 (9.6)	217 (16.7)
**CVA**	1849 (14.7)	27 (15.5)	224 (15.8)	585 (14.6)	771 (13.8)	228 (17.5)
**AAA**	356 (2.8)	2 (1.1)	15 (1.1)	124 (3.1)	163 (2.9)	49 (3.8)
**Systolic blood pressure (mm/Hg)**	140.7 ± 21.6	121.7 ± 10.9	125.0 ± 13.9	136.5 ± 20.5	146.6 ± 21.5	148.4 ± 19.6
**Diastolic blood pressure (mm/Hg)**	82.8 ± 12.5	73.3 ± 7.9	75.3 ± 58.8	80.5 ± 11.7	85.6 ± 12.6	86.9 ± 12.4
**Pulse pressure (mm/Hg)**	57.9 ± 15.6	48.4 ± 9.1	49.7 ± 10.7	55.89 ± 15.0	60.9 ± 16.3	61.4 ± 15.2
**Antihypertensive prescription**	5822 (46.1)	0 (0)	95 (6.7)	1319 (33.0)	3486 (62.4)	887 (68.2)
**Hypertension**	8391 (66.5)	0 (0)	157 (11.0)	1973 (49.4)	4889 (87.5)	1300 (100)
**Total cholesterol (mmol/L)**	5.1 ± 1.4	3.6 ± 0.5	4.8 ± 1.5	5.1 ± 1.4	5.2 ± 1.3	5.4 ± 1.4
**Low-density lipoprotein cholesterol (mmol/L)**	3.1 ± 1.2	1.8 ± 0.4	2.9 ± 1.3	3.1 ± 1.2	3.1 ± 1.1	3.2 ± 1.1
**High-density lipoprotein cholesterol (mmol/L)**	1.3 ± 0.4	1.5 ± 0.5	1.4 ± 0.4	1.3 ± 0.4	1.2 ± 0.4	1.1 ± 0.3
**Triglycerides (mmol/L)**	1.8 ± 1.8	0.8 ± 0.3	1.2 ± 0.6	1.6 ± 1.3	1.9 ± 2.0	2.5 ± 2.9
**Lipid lowering medication prescription**	5425 (43)	0 (0)	374 (26.3)	1578 (39.5)	2753 (49.3)	709 (54.5)
**Dyslipidemia**	11488 (91.1)	0 (0)	1025 (72.1)	1973 (49.4)	5516 (98.7)	1300 (100)
**Current smoking**	3544 (28.1)	0 (0)	67 (4.7)	687 (17.2)	1465 (26.2)	1300 (100)
**Diabetes**	2302 (18.2)	28 (16.1)	149 (10.5)	580 (14.5)	1229 (22.0)	287 (22.1)
**eGFR (ml/min/1.73 m^2^)**	79.9 ± 19.5	89.4 ± 21.8	83.3 ± 17.7	79.9 ± 18.9	78.2 ± 19.7	81.9 ± 21.1

For continuous variables of baseline characteristics, mean ± SD was calculated. For categorical variables, frequencies and percentages were calculated. SES, socioeconomic status; CAD, coronary artery disease; PAD, peripheral artery disease; CVA, cerebrovascular accidents; AAA, abdominal aortic aneurysm; Hypertension, systolic blood pressure ≥140 mmHg and/or diastolic blood pressure ≥90 mmHg and/or prescribed antihypertensive drugs; Dyslipidemia, non-HDL levels (total cholesterol minus HDL-C) >2.6 mmol/L and/or the prescription of lipid lowering drugs; MDRD-clearance, modification of diet in renal disease.

### Prevalence of clusters

Overall, 11.4% of the patients had one risk factor, 32.0% had two risk factors, 44.8% had three risk factors, and 10.4% had all four risk factors (Table 2). Dyslipidemia was the most prevalent individual risk factor (91.4%). The hypertension-dyslipidemia cluster was the most common cluster with two risk factors (62%). The hypertension-dyslipidemia-overweight cluster was the most common cluster with three risk factors (43.4%). The prevalence of all the four risk factors (smoking-hypertension-dyslipidemia-overweight) was 10.4% (Tables 2 and 3).

**Table 2:** The relation with CVD events, prevalence, and PAF (%) of the four risk factor levels.

Number of risk factors	HR (95% CI)	Prevalence	PAF, %

**0**	Reference	1.4	Reference
**1**	1.65 (0.77; 3.54)	11.4	6.9
**2**	2.61 (1.24; 5.50)	32.0	34.0
**3**	3.25 (1.55; 6.84)	44.8	50.1
**4**	3.74 (1.77; 7.93)	10.4	22.2

Reference group are patients (N = 174) with none of the four risk factors. Prevalence per level was calculated as number of patients in that cluster level divided by the total amount of patients analyzed (n = 12,479). PAF calculation was included in the method section.

**Table 3:** The relation with CVD events, prevalence and, PAF (%) of individual risk factors and the clusters.

Overall			

	HR (95% CI)	Prevalence	PAF,%

**None of the four risk factors**	Reference	1.4	Reference
**Current smoking**	2.41 (0.81; 7.17)	28.2	28.4
**Hypertension**	1.30 (0.53; 3.18)	66.6	16.7
**Dyslipidemia**	1.25 (0.57; 2.70)	91.4	18.6
**Overweight**	1.78 (0.75; 4.23)	65.1	33.7
**Smoking – Hypertension**	3.17 (1.15; 8.74)	17.1	27.1
**Smoking – Dyslipidemia**	2.35 (1.08; 5.13)	27.1	26.8
**Smoking – Overweight**	1.11 (0.23; 5.36)	17.3	1.9
**Hypertension – Dyslipidemia**	1.95 (0.92; 4.14)	62	37.1
**Hypertension – Overweight**	1.40 (0.62; 3.17)	46.6	15.7
**Dyslipidemia – Overweight**	1.70 (0.80; 3.61)	60.6	29.8
**Smoking – Hypertension – Dyslipidemia**	4.06 (1.91; 8.63)	16.4	33.4
**Smoking – Hypertension – Overweight**	2.51 (0.93; 6.73)	11	14.2
**Smoking – Dyslipidemia – Overweight**	2.86 (1.33; 6.16)	16.4	23.4
**Hypertension – Dyslipidemia – Overweight**	1.99 (0.94; 4.18)	43.4	30.1
**Smoking – Hypertension – Dyslipidemia – Overweight**	3.25 (1.53; 6.88)	10.4	19.0

Reference group are patients (N = 174) with none of the four risk factors. Prevalence for each risk factor and cluster was calculated as number of patients in that cluster divided by the total amount of patients analyzed (n = 12,479), adding up the prevalence of clusters that also contain that specific risk factor combination (e.g. the 91.4% for L was based on L presence in the L individual risk factor, SL, LO, SHL, SLO, HLO and SHLO clusters). The PAF calculation was included in the method section.

### Relation between risk factor clusters and cardiovascular events

During a mean follow-up period of 8.0 ± 5.2 years, 1836 CVD events and all-cause mortality (14.7%) were registered.

**Figure 1:**
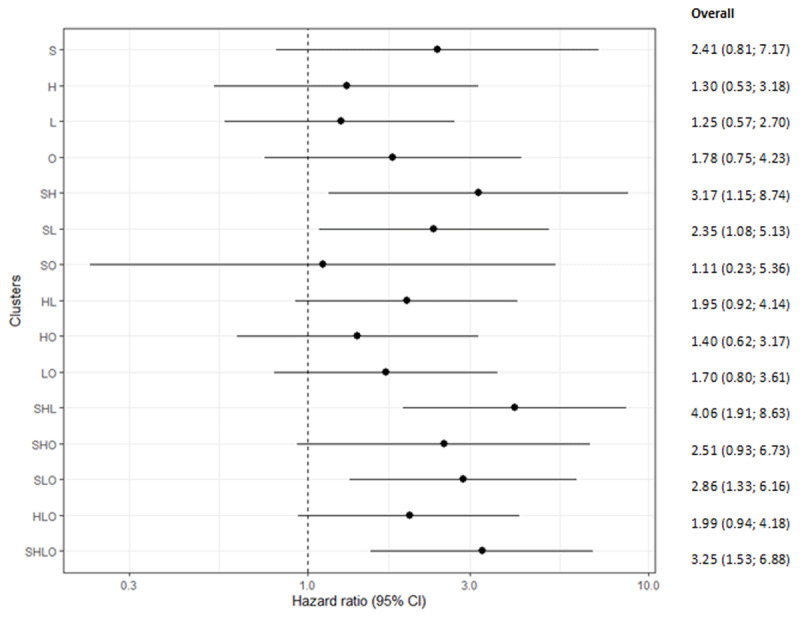
Hazard ratio and its corresponding 95% CI (on the log-scale) for individual risk factors and risk factor clusters adjusted for age and sex. A no difference in CVD risk between cluster and the reference group giving a value of one is displayed in the plot. Reference group are patients (N = 174) with none of the four risk factors. L, dyslipidemia; S, current smoking; H, hypertension; O, overweight.

Among patients with one risk factor, smoking was associated with the highest HR of CVD and all-cause mortality: 2.41 (0.81; 7.17). Among patients with two risk factors, the smoking-hypertension cluster had the highest HR: 3.17 (1.15; 8.74). For clusters with three risk factors, the smoking-hypertension-dyslipidemia combination had the highest HR: 4.06 (1.91; 8.63). The HRs in patients with all four risk factors was 3.25 (1.53; 6.88) (Figure 1, Table 3).

**Figure 2:**
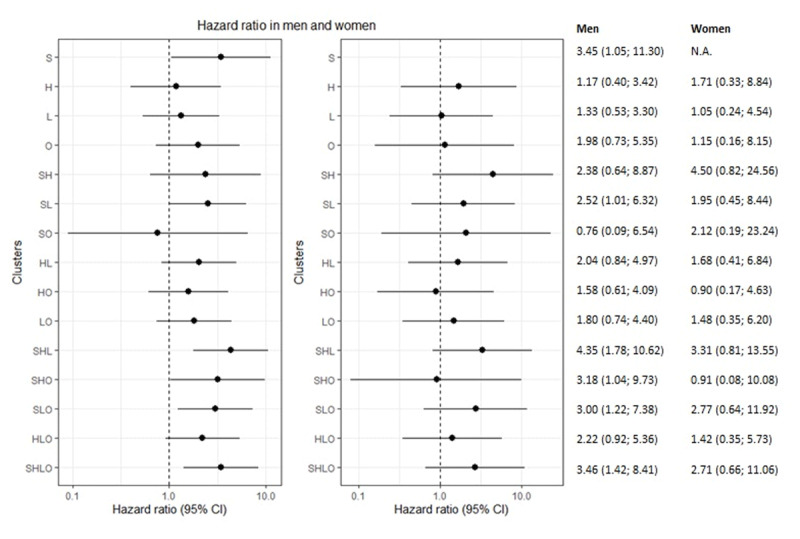
Hazard ratios and its corresponding 95% CIs (on the log scale) by sex. A no difference in CVD risk between clusters and the reference group giving a value of 1 is displayed in the plot. Reference group are patients (N = 174) with none of the four risk factors. L, dyslipidemia; S, current smoking; H, hypertension; O, overweight.

**Figure 3:**
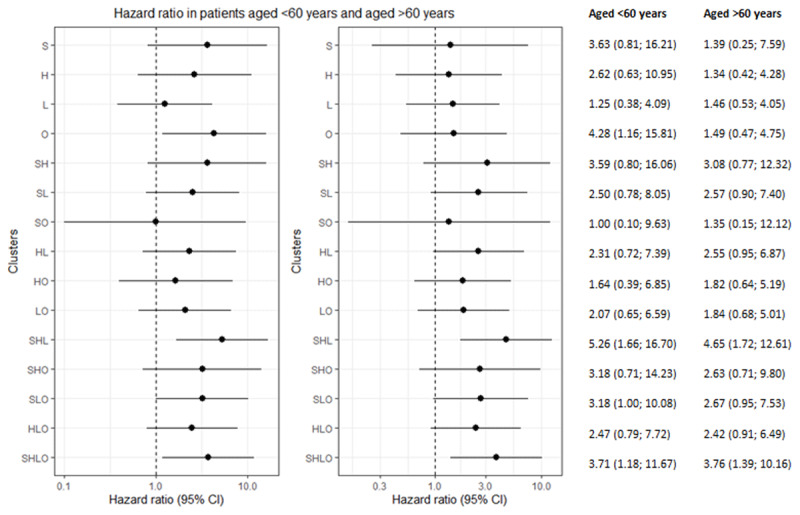
Hazard ratios and its corresponding 95% CIs (on the log scale) by age. A no difference in CVD risk between clusters and the reference group giving a value of 1 is displayed in the plot. Reference group are patients (N = 174) with none of the four risk factors. L, dyslipidemia; S, current smoking; H, hypertension; O, overweight.

Analyses stratified by sex showed that smoking was the strongest individual risk factor in both men and hypertension in women (Figure 2, Table 4). For those with two risk factors, the smoking-dyslipidemia was the strongest cluster in men and the smoking-hypertension cluster in women. For both men and women with three risk factor clusters, the smoking-hypertension-dyslipidemia cluster was associated with the highest risk of CVD. Men with all risk factors had a HR of 3.46 (1.42; 8.41) and women a HR of 2.71 (0.66; 11.06). Analyses stratified by age showed that overweight was the strongest risk factor for both younger and older patients with one risk factor. The smoking-hypertension and smoking-hypertension-dyslipidemia cluster were associated with the highest HR in both younger and older patients with two or three risk factors. The HR among patients with all risk factors was 3.71 (1.18; 11.67) for those aged <60 years and 3.76 (1.39; 10.16) for those aged > 60 years (Figure 3, Table 4).

**Table 4:** The relation with CVD events, prevalence and PAF (%) of individual risk factors and the clusters stratified by sex and age.

	Men (n = 8187)			Women (n = 4292)		

	HR (95% CI)	Prevalence	PAF,%	HR (95% CI)	Prevalence	PAF,%

**None of the four risk factors**	Reference	1.2	Reference	Reference	1.7	Reference
**Smoking**	3.45 (1.05;11.30)	29.0	41.5	N.A.	26.9	N.A.
**Hypertension**	1.17 (0.40; 3.42)	65.5	10.0	1.71 (0.33; 8.84)	68.3	32.7
**Dyslipidemia**	1.33 (0.53; 3.30)	68.4	18.4	1.05 (0.24; 4.54)	91.9	4.4
**Overweight**	1.98 (0.73; 5.35)	68.7	40.2	1.15 (0.16; 8.15)	58.8	8.1
**Smoking – Hypertension**	2.38 (0.64; 8.87)	17.1	19.1	4.50 (0.82; 24.56)	17	37.3
**Smoking – Dyslipidemia**	2.52 (1.01; 6.32)	27.1	29.2	1.95 (0.45; 8.44)	24.9	19.1
**Smoking – overweight**	0.76 (0.09; 6.54)	18.4	0	2.12 (0.19; 23.24)	14.1	13.6
**Hypertension – Dyslipidemia**	2.04 (0.84; 4.97)	60.3	38.5	1.68 (0.41; 6.84)	63.4	30.1
**Hypertension – Overweight**	1.58 (0.61; 4.09)	48.1	21.8	0.90 (0.17; 4.63)	44.1	0
**Dyslipidemia – Overweight**	1.80 (0.74; 4.40)	63.1	33.5	1.48 (0.35; 6.20)	54.9	20.9
**Smoking – Hypertension – Dyslipidemia**	4.35 (1.78; 10.62)	16.1	35.0	3.31 (0.81; 13.55)	15.8	26.7
**Smoking – Hypertension – Overweight**	3.18 (1.04; 9.73)	11.6	20.2	0.91 (0.08; 10.08)	9.8	0
**Smoking – Dyslipidemia – Overweight**	3.00 (1.22; 7.38)	17.4	25.8	2.77 (0.64; 11.92)	13.4	19.1
**Hypertension – Dyslipidemia – Overweight**	2.22 (0.92; 5.36)	44.5	35.2	1.42 (0.35; 5.73)	41.3	14.8
**Smoking – Hypertension – Dyslipidemia – Hypertension**	3.46 (1.42; 8.41)	11	21.3	2.71 (0.66; 11.06)	9.3	13.7
	**Age < 60** **(n = 7398)**			**Age > 60 (n = 5081)**		

	**HR (95% CI)**	**Prevalence**	**PAF,%**	**HR (95% CI)**	**Prevalence**	**PAF,%**

**None of the four risk factors**	Reference	1.6	Reference	Reference	1.1	Reference
**Smoking**	3.63 (0.81; 16.21)	34.6	47.6	1.39 (0.25; 7.59)	18.9	6.9
**Hypertension**	2.62 (0.63; 10.95)	60.0	49.3	1.34 (0.42; 4.28)	76.3	20.6
**Dyslipidemia**	1.25 (0.38; 4.09)	92.4	18.8	1.46 (0.53; 4.05)	86.5	28.5
**Overweight**	4.28 (1.16; 15.81)	64.0	67.7	1.49 (0.47; 4.75)	63.0	23.6
**Smoking – Hypertension**	3.59 (0.80; 16.06)	19.1	33.1	3.08 (0.77; 12.32)	14.1	22.7
**Smoking – Dyslipidemia**	2.50 (0.78; 8.05)	32.3	32.6	2.57 (0.90; 7.40)	17.5	21.6
**Smoking – overweight**	1.00 (0.10; 9.63)	21.2	0	1.35 (0.15; 12.12)	10.6	3.6
**Hypertension – Dyslipidemia**	2.31 (0.72; 7.39)	56.2	42.4	2.55 (0.95; 6.87)	70.4	52.2
**Hypertension – Overweight**	1.64 (0.39; 6.85)	42.6	21.4	1.82 (0.64; 5.19)	52.4	30.1
**Dyslipidemia – Overweight**	2.07 (0.65; 6.59)	60.1	39.1	1.84 (0.68; 5.01)	56.8	32.3
**Smoking – Hypertension – Dyslipidemia**	5.26 (1.66; 16.70)	17.9	43.3	4.65 (1.72; 12.61)	13.7	33.3
**Smoking – Hypertension – Overweight**	3.18 (0.71; 14.23)	12.9	21.9	2.63 (0.71; 9.80)	8.2	11.8
**Smoking – Dyslipidemia – Overweight**	3.18 (1.00; 10.08)	20.2	30.6	2.67 (0.95; 7.53)	9.9	14.2
**Hypertension – Dyslipidemia – Overweight**	2.47 (0.79; 7.72)	40.3	37.2	2.42 (0.91; 6.49)	48.1	40.6
**Smoking – Hypertension – Dyslipidemia – Hypertension**	3.71 (1.18; 11.67)	12.3	25.0	3.76 (1.39; 10.16)	7.7	17.5

Reference group are patients (N = 174) with none of the four risk factors. Prevalence for each risk factor and cluster was calculated as number of patients in that cluster divided by the total amount of patients analyzed in that sub-group, adding up the prevalence of clusters that also contain that specific risk factor combination (e.g. the 60.3% for HL in men was based on HL presence in the HL cluster, SHL, HLO and SHLO clusters). The PAF calculation was included in the method section.

### PAFs of individual risk factors and risk factor clusters

Overall, overweight was associated with the highest PAF of 33.7%, the hypertension-dyslipidemia among two risk factors, PAF: 37.1%, and the smoking-hypertension-dyslipidemia among three risk factors, PAF: 33.4%. The PAF of those with all four risk factors was 19.0% (Table 3). The hypertension-dyslipidemia cluster had the highest PAFs among men, PAF: 38.5%. Among women, the smoking-hypertension cluster had the highest PAFs, 37.3%. Concerning patients aged <60 years, the smoking-hypertension-dyslipidemia cluster had the highest PAF, 43.3%. The hypertension-dyslipidemia cluster had the highest PAF in patients aged > 60 years, PAF: 52.2%.

## Discussion

The present study among patients at high risk of CVD examined the prevalence of risk factor clusters and their relation with cardiovascular events and all-cause mortality. We show that the co-existence of a greater number of risk factors was associated with a greater risk of CVD and all-cause mortality. The strength of the association differed across and within risk factor clusters. The smoking-hypertension-dyslipidemia cluster was associated with the highest CVD risk and all-cause mortality and the hypertension-dyslipidemia cluster had the highest population attributable fraction. Our results also showed that the smoking-hypertension cluster was associated with a higher risk of CVD and all-cause mortality compared to the smoking-hypertension-overweight cluster. This was also the case for the smoking-hypertension-dyslipidemia cluster, which was associated with a higher risk of CVD and all-cause mortality compared to the smoking-hypertension-dyslipidemia-overweight cluster, which is contrary to common sense. This could potentially be explained by the fact that clusters including the smoking and overweight had a relatively low prevalence, that is, a combination of these two risk factors is not typically seen in patients, compared to the other clusters.

Studies on MetS, defined as the co-existence of three or more metabolic abnormalities, have demonstrated that MetS is associated with an increased risk of cardiovascular events and all-cause mortality in patients with coronary artery disease, cerebrovascular disease, peripheral artery disease and abdominal aortic aneurysm [13].

This is the first study to assess the association of risk factor clusters with CVD and all-cause mortality in a high-risk population comprising patients with either risk factors for arterial disease or with symptomatic arterial disease. Previous studies in the general population have shown that combinations of CVD risk factors are related to an increased CVD risk [10,20]. A study including 47,385 individuals compared the prevalence and clustering of major cardiovascular risk factors between adults in the Netherlands and China [20]. The study showed that cardiovascular risk profiles and clustering patterns differ between the Dutch and the Chinese as well as between men and women [20]. As such, insights in clustering patterns within a specific population are needed to effectively prevent and manage CVD [8,20,21]. Results from the Asia-Pacific Cohort Studies Collaboration (APCSC), including 314,024 individuals from 44 studies and over 6,200 CVD events showed that clusters of major modifiable risk factors act likewise on the risk of CVD in Asian and Caucasian populations. Risk factor clusters including hypertension were associated with the highest excess risk of CVD [10]. In both Caucasian populations and SMART, clusters including dyslipidemia were most prevalent (60.9% vs. 91.4%) whereas clusters including hypertension and smoking were associated with the highest CVD risk (HR, 3.81 (2.85; 5.09) vs. 4.06 (1.91; 8.63) [10]. The hypertension-dyslipidemia cluster had the highest PAF in both Caucasian populations from the APCSC and SMART (29.0% vs. 37.1%) [10].

The similarities between the findings in APCSC and SMART illustrate that approaches to reduce the burden of the most prevalent clusters and clusters associated with the highest CVD risk should target the same risk factors, using a combination of lifestyle and therapeutic interventions.

Since high-risk patients generally have co-existing risk factors, targeting multiple risk factors for a moderate decline could be at least as effective as extensive reductions in an individual risk factor. Previous studies have confirmed the clustering of cardiovascular risk factors has more harmful cardiovascular effects than a single risk factor [22,23]. Also, the substantial variation in CVD risk associated with different risk factor clusters in high-risk patients indicates the importance of individualized management of cardiovascular risk by focusing on the patient’s risk factor profile through lifestyle changes and treatment for all risk factors in question [24]. Prioritizing clusters, by targeting risk factors associated with the highest risk and prevalence may be an appropriate approach when the resources and ability to address all risk factors are limited, especially in lower-middle income countries. Results were broadly similar by sex and age group. Previous studies have shown that increasing age and being a male are associated with the presence of more risk factors [20,23]. However, due to relatively low prevalence and events in certain clusters, this study was unable to find these conclusive differences between the subgroups.

In addition to pharmaceutical interventions and individual lifestyle changes, policy interventions could effectively lower the prevalence of risk factors and the subsequent risk of CVD. As an example, the smoking legislation has shown to successfully lower the risk of hospitalization and death in smoking related cerebrovascular, cardiac and respiratory diseases [25]. As a result of policy interventions, the entire population can benefit from improved health status, that is, this fits in the one-size-benefits-all approach.

A large base of evidence shows the advantages of smoking cessation on overall CV risk, as it has substantially a greater magnitude compared to the effect of medical treatment on a single major risk factor [16]. Smoking cessation should therefore be a primary goal in patients with(out) vascular disease [17]. Hypertension also needs serious attention. A combination of blood pressure-lowering medication in patients not yet treated or not reaching their target level and behavioral and lifestyle changes could effectively reduce the growing worldwide burden associated with high systolic blood pressure [26]. When resources are limited, like in low-and-middle income countries, an alternative would be to opt for statin treatment as this is relatively inexpensive and because smoking cessation and controlling blood pressure can be very time-consuming. Furthermore, controlling blood pressure includes the usage of multiple pills and various outpatient clinics visits. Salt reduction was also found to be a very cost-effective method for improving health outcomes, mainly in individuals with hypertension [27].

Strengths of this study include the prospective design, well characterized cohort with nearly complete information on risk factor levels, and the long follow-up duration. A limitation of this study is the relatively low prevalence and limited number of events in certain clusters. This limited our ability to find conclusive differences in the impact of risk factor clusters, especially in the subgroup analyses. Most risk estimates were not statistically significant, which is likely to be due to the low prevalence and limited number of events in certain clusters. Another limitation is that clusters with diabetes were not included due to the relatively low prevalence.

To conclude, clusters including smoking and hypertension contributed to the highest risk of CVD and all-cause mortality. This study confirms that risk factor clustering is common among patients at high-risk for CVD and is associated with an increased risk of CVD and all-cause mortality.

## Data Accessibilty Statement

Requests for using the information can be directed to prof dr. F.L.J. Visseren, chairman of the UCC-SMART study. When the request has been approved by the UCC-SMART study group study data will be made available to the applicant, taking Dutch regulatory and ethical regulations into account.

## Appendix: UCC-SMART study group members

F.W. Asselbergs & H.M. Nathoe, Department of Cardiology;

G.J. de Borst, Department of Vascular Surgery;

M.L. Bots and M.I. Geerlings, Julius Center for Health Sciences and Primary Care;

M.H. Emmelot-Vonk, Department of Geriatrics;

P.A. de Jong and T. Leiner, Department of Radiology;

A.T. Lely, Department of Gynecology and Obstetrics;

N.P. van der Kaaij, Department of Cardiothoracic Surgery;

L.J. Kappelle and Y.M. Ruigrok, Department of Neurology;

M.C. Verhaar, Department of Nephrology & Hypertension;

F.L.J. Visseren (chair) and J. Westerink, Department of Vascular Medicine,

All University Medical Center Utrecht and Utrecht University.
